# Betacoronavirus Assembly: Clues and Perspectives for Elucidating SARS-CoV-2 Particle Formation and Egress

**DOI:** 10.1128/mBio.02371-21

**Published:** 2021-09-28

**Authors:** David Bracquemond, Delphine Muriaux

**Affiliations:** a Institut de Recherche en Infectiologie de Montpellier, Université de Montpellier, CNRS UMR9004, Montpellier, France; CIRI, International Center for Infectiology Research; Albert Einstein College of Medicine

**Keywords:** SARS-CoV-2, viral assembly, egress

## Abstract

In 2019, a new pandemic virus belonging to the betacoronavirus family emerged, severe acute respiratory syndrome coronavirus 2 (SARS-CoV-2). This new coronavirus appeared in Wuhan, China, and is responsible for severe respiratory pneumonia in humans, namely, coronavirus disease 2019 (COVID-19). Having infected almost 200 million people worldwide and caused more than 4.1 million deaths as of today, this new disease has raised a significant number of questions about its molecular mechanism of replication and, in particular, how infectious viral particles are produced. Although viral entry is well characterized, the full assembly steps of SARS-CoV-2 have still not been fully described. Coronaviruses, including SARS-CoV-2, have four main structural proteins, namely, the spike glycoprotein (S), the membrane glycoprotein (M), the envelope protein (E), and the nucleocapsid protein (N). All these proteins have key roles in the process of coronavirus assembly and budding. In this review, we gathered the current knowledge about betacoronavirus structural proteins involved in viral particle assembly, membrane curvature and scission, and then egress in order to suggest and question a coherent model for SARS-CoV-2 particle production and release.

## INTRODUCTION

Coronaviruses are small enveloped RNA viruses of around 100 nm in diameter, containing a single-stranded positive-sense RNA of 30 kb on average, which can infect animals and humans. In 2019, the emergence of severe acute respiratory syndrome coronavirus 2 (SARS-CoV-2) caused a worldwide pandemic leading to coronavirus disease 2019 (COVID-19) in humans. A notable amount of work has been carried out on the entry and relationship between the spike protein and the main host human receptor, angiotensin-converting enzyme 2 (ACE2), but so far, few articles have been published focusing on SARS-CoV-2 particle assembly and egress within host cells. Overall, genome similarities exist between SARS-CoV-2 and other betacoronaviruses, reaching 70% in the case of Middle East respiratory syndrome coronavirus (MERS-CoV) and 83% for severe acute respiratory syndrome coronavirus 1 (SARS-CoV-1), responsible for the 2012 and 2002–2003 epidemic outbreaks in Saudi Arabia and China, respectively. When comparing only structural proteins, similarities with SARS-CoV-2 drop to 30 to 50% for MERS-CoV while reaching 85% for SARS-CoV-1 (1). Another well-known virus belonging to the betacoronavirus family that can be compared with SARS-CoV-2 is murine hepatitis virus (MHV), which has undergone in-depth studies, facilitated by the fact that it is not a human pathogen. Along these lines, and throughout this review, we mostly compare MHV and SARS-CoV-1 with recent studies on SARS-CoV-2 assembly. SARS-CoV-2, like other coronaviruses, encodes 19 proteins, including 4 main structural proteins, namely, the spike (S), membrane (M), envelope (E), and nucleocapsid (N) proteins (2). Particle assembly and budding occur in the endoplasmic reticulum (ER)-Golgi intermediate compartment (ERGIC), and the newly formed viral particles are then released at the cell plasma membrane through unclear trafficking pathways (3–5). In this review, we mainly go over the late steps of coronavirus replication that lead to the assembly of new viral particles, starting with the location of viral RNA synthesis and its incorporation into the newly formed virions. We then describe the interactions between the structural proteins, their intracellular location, and their role in the assembly of new virions and in viral particle egress.

## VIRAL RNA REPLICATION OCCURS IN DOUBLE-MEMBRANE VESICLES

Double-stranded viral RNA, an intermediate in viral RNA synthesis, has been identified in double-membrane vesicles (DMVs), which seem to be either completely closed or partially open and are connected to the endoplasmic reticulum (ER) (3, 6). DMVs seem to be the only place where viral RNA synthesis occurs despite the presence of other conceivable replication organelles such as convoluted membranes and single-membrane vesicles (SMVs), independently of the cell line or the type of coronavirus (6). This spatial compartmentalization of the transcription process could help evade the innate immune system, i.e., remaining unrecognizable by the pattern recognition receptors (3, 7). The precise mechanism by which DMVs are created is currently unknown, but several nonstructural or accessory proteins may be involved in the process, as reported for SARS-CoV-1 (8). Indeed, SARS-CoV-1 nonspecific protein 3 (nsp3) and nsp4 are responsible for zippered membranes with a maze-like body (MLB), interconnected with the ER. The addition of nsp6 leads to DMV-like structures, although they are smaller than those in infected cells, while MLBs can still be observed. Other coronavirus proteins could be involved, such as nsp10, known to keep nsp4 and nsp6 in the vicinity, which allows the efficient production of DMVs. In addition, the viral RNA might impact DMV size (8). As for SARS-CoV-2, a study pointed out that the accessory protein Orf3a increases the amount of double-membrane autophagosomes while decreasing their maturation in autolysosomes. This observation raises the question of using this membrane reservoir for viral replication and as a way to hide from the immune system. This might be a partial answer in determining DMV origins in the case of SARS-CoV-2 (9). For SARS-CoV-2, the DMVs were very well characterized thanks to recent high-resolution microscopy techniques: DVMs are composed of two membranes separated by 18-nm luminal spacing, with an average diameter of 340 nm for the inner membrane, similar to SARS-CoV-1 DMVs (7). As for DMVs induced by MHV, a murine coronavirus, they appear smaller, with an average diameter of 260 nm (10), revealing that DMVs might slightly differ from one host to another, probably depending on virus-host adaptation. Inside these structures, RNA synthesis occurs discontinuously. The transcription complex synthesizes the full-length viral RNA but also small pieces of subgenomic RNA (2).

## THE VIRAL GENOMIC RNA LEAVING DMVs FOR ASSEMBLY

For the coronaviruses SARS-CoV-2 and MHV, it is well known that the viral RNA leaves DMVs through membrane pores and is condensed with the cytosolic nucleocapsid protein N. However, a striking difference exists between SARS-CoV-2 and MHV concerning these pores: only 9 have been identified out of the 24 DMVs for the first virus, compared to an average of 8 per DMV for the second (3). The limited number of such outlets calls into question the existence of other pathways allowing the newly synthesized RNA to be transported out of the inner membrane of DMVs. Nonetheless, these pores have been characterized in MHV infectious cells using cryo-electron tomography (cryo-ET), showing a 6-fold symmetry structure embedded between the inner and the outer membranes of DMVs, with a canal of 2 to 3 nm in diameter at the tightest point, ensuring RNA passage. The cytoplasmic part, which has a crown-like structure, is mainly composed of nsp3. As for the rest of the pore, nsp4 and nsp6 are good candidates thanks to their transmembrane domains (TMDs) and their probable role in DMV formation itself. When analyzing their data, Wolff et al. hypothesized that a protein inside the DMVs would guide the viral RNA through these pores, probably one of the RNA-dependent RNA polymerase (RdRp) complex proteins (10), and suggested that it might be the same for SARS-CoV-2.

## THE NUCLEOCAPSID PROTEIN N AND VIRAL RNA PACKAGING

**FIG 1:**
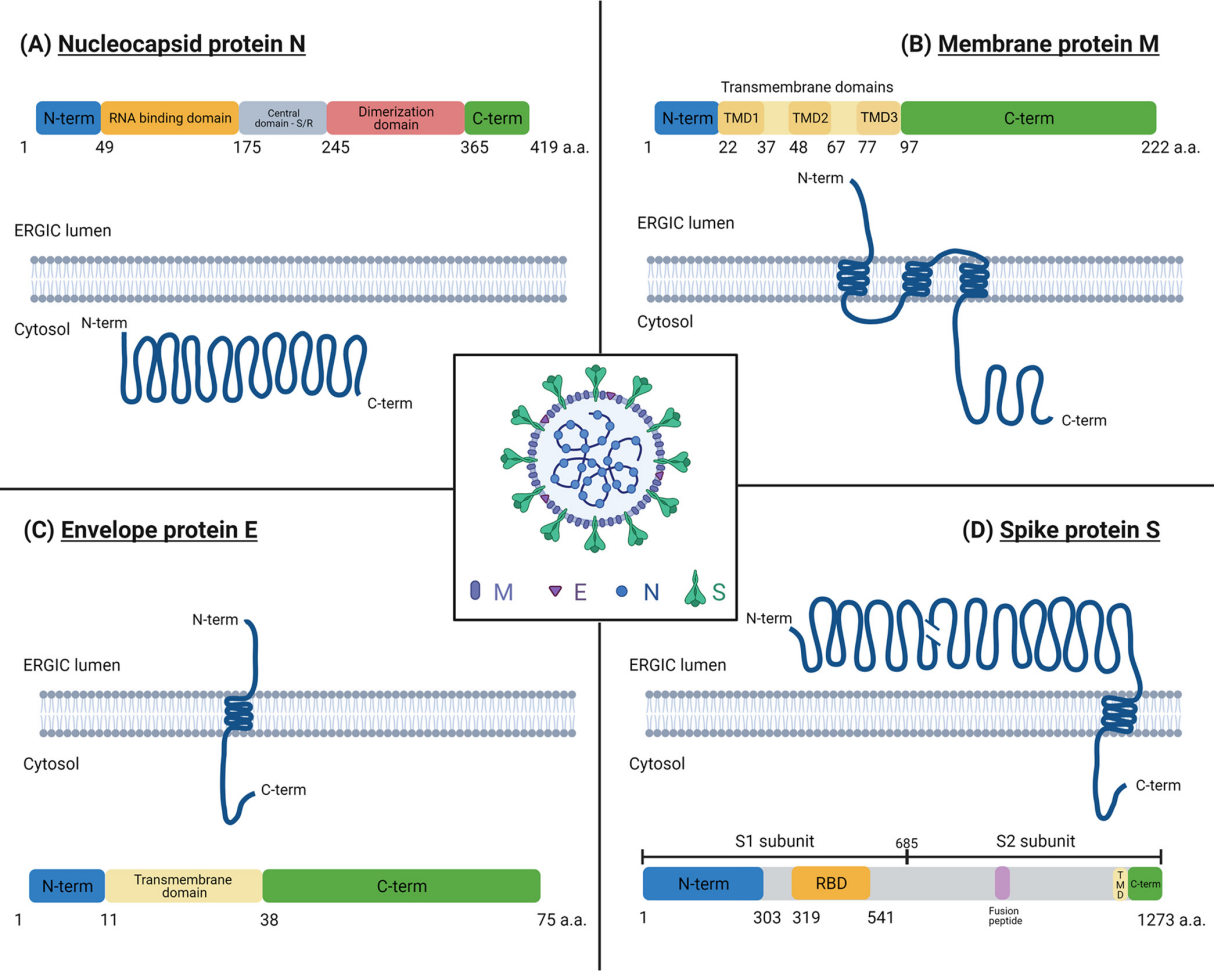
Schemes and membrane topology of the structural SARS-CoV-2 proteins nucleoprotein (N) (A), membrane (M) (B), viroporin envelope (E) (C), and spike (S) (D). N-term, amino terminus; C-term, carboxy terminus; ERGIC, endoplasmic reticulum-Golgi intermediate compartment; a.a., amino acids; S/R, serine/arginine; TMD, transmembrane domain; RBD, receptor binding domain. A viral particle with the schematic location of the M, N, E, and S viral proteins is shown in the center. (Adapted from references 12 and 24 with the help of BioRender [2020].)

The SARS-CoV-2 nucleocapsid protein N is a cytosolic protein of 46 kDa consisting of 419 amino acids, with 85% conserved identity with SARS-CoV-1 (1). SARS-CoV-2 N is composed of different domains (Fig. 1A), each having a specific role: the amino-terminal (N-terminal) region, which likely interacts with the viral RNA packaging signal; an RNA binding region that chaperones the viral RNA; a serine/arginine-rich region; a central region implicated in phase separation; and, finally, the carboxy-terminal (C-terminal) region involved in N dimerization and N-M interactions (from the N terminus to the C terminus) (11, 12). A domain in N with high binding affinity for the viral RNA might be the origin of N-RNA complexation, whereas the rest of N seems to have less affinity for the remainder of the strand (11). This is in agreement with a study on SARS-CoV-1 where N is noted as being essential for viral RNA packaging (13).

**FIG 2:**
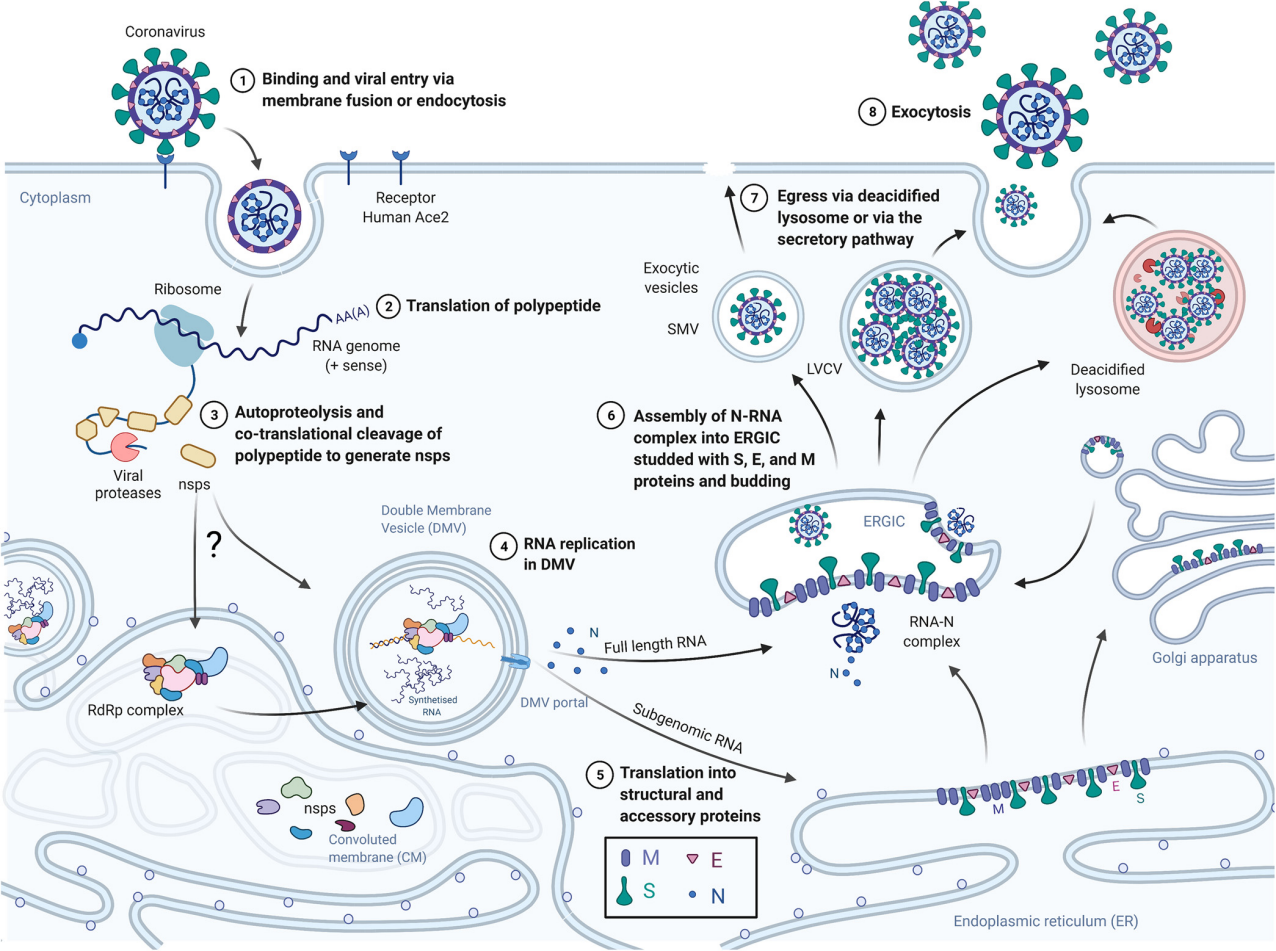
Proposed model of SARS-CoV-2 replication focused on virus assembly and egress. Steps 1, 2, 3, and 4 represent the early phases of the viral replication cycle, with entry and RNA replication, while the focus of this review is on the late phases, i.e., steps 6, 7, and 8, which represent the assembly of the N-RNA complexes with M, E, and S proteins at the ERGIC toward several exit routes in small, large secretory, or deacidified lysosomal vesicles before plasma membrane exocytosis. ERGIC, endoplasmic reticulum-Golgi intermediate compartment; nsps, nonstructural proteins; RdRp, RNA-dependent RNA polymerase; SMV, single-membrane vesicle; LVCV, large virus-containing vesicle. (Adapted from “Life Cycle of Coronavirus” by BioRender [https://app.biorender.com/biorender-templates] [2020].)

For MHV, N was reported to be located near the pores’ exit, meaning that the viral RNA may immediately be complexed by N as it exits the DMVs (Fig. 2); a similar mechanism for SARS-CoV-2 has been suggested. This raises a question about the complexation and the incorporation of only the full-length 30-kb viral RNA or all synthesized RNAs, including subgenomic viral RNAs, which represent about 95% of the *de novo* RNA (10). Indeed, the incorporation of the N-RNA complex itself is likely mediated by a packaging signal, which has not yet been identified in SARS-CoV-2 but is well known in other coronaviruses such as SARS-CoV-1 or MHV (11, 14). Even though a specific RNA packaging signal should exist in SARS-CoV-2, it needs to be confirmed: a first study highlights RNA stem-loops in the 5′ and 3′ untranslated regions (UTRs) (15). On the other hand, mRNA incorporation into virions should also be considered during particle assembly. For instance, in MHV, one cellular mRNA is able to interact with M and thus be incorporated into the particle (16). The main difference between MHV and SARS-CoV-1 is that N is not required for the former in order to form the virion, which is probably why an interaction between the M protein and the RNA of MHV exists. Nevertheless, an interaction between M and the viral RNA, and, therefore, the incorporation of mRNA, cannot be excluded, even though it seems very unlikely; thus, more studies need to be carried out (11). This leaves an open question regarding the role of nonviral RNA in particle assembly.

## N-RNA COMPLEX READY FOR ASSEMBLY

In SARS-CoV-2, the N-RNA complex seems to be organized as numerous subcomplexes of around 12 N proteins, overlaying 800 nucleotides, together forming a G shape, also known as ribonucleoproteins (RNPs). The succession of these RNPs along the 30-kb viral RNA leads to a beads-on-a-string structure (17). Several N domains were reported to be involved in the condensation of N with the viral RNA, particularly the central region, suggesting a collective contribution of N domains. However, the number of N proteins per complex is still uncertain (11). All these RNPs, estimated at 30 to 35 per particle using cryo-ET images, are well ordered, in either hexagonal or tetrahedron distributions, with a more prominent population of the first kind (11, 17). While SARS-CoV-2 N self-associates to form tetramers, the presence of viral RNA causes the full-length N protein to assemble into large oligomers, likely due to protein-protein interactions between different N domains or through RNA-N interactions (18). Furthermore, it was recently described for SARS-CoV-2 that the central domain and RNA binding region of N are responsible for phase separation with the viral RNA. That behavior is regulated by the phosphorylation of the serine/arginine-rich region of N, which is highly phosphorylated in infected cells (11, 15, 18, 19). Indeed, it is during the packaging process that the N-RNA complex undergoes this phase separation from liquid-like to gel-like condensates due to N-RNA interactions. The condensation process then continues when N interacts with the membrane protein M (11). On the other hand, the N-RNA complex keeps its liquid-like condensation state when N is highly phosphorylated (19). Together, these results underline an unclear double role of N in N-RNA incorporation into particles (gel-like condensate) and its potential participation in the transcription process near the DMVs (liquid-like condensate) (11, 19).

In conclusion, viral RNA packaging starts with the complexation of the viral RNA by the nucleocapsid N proteins. How the following incorporation of the N-RNA complex into the particle occurs remains unclear for SARS-CoV-2, but previous studies on other betacoronaviruses show that all RNPs will interact preferentially with the membrane protein M, likely through direct N-M interactions via their carboxy-terminal tails as for SARS-CoV-1 (20).

## THE M PROTEIN IS A KEY COMPONENT FOR CORONAVIRUS ASSEMBLY

The coronavirus transmembrane glycoprotein M is a 222-amino-acid protein of 25 kDa with a triple-helix bundle shape (12, 21). Along with N, it is one of the most abundant viral proteins and plays a crucial role in viral particle assembly because of key interactions with itself and other structural proteins. M has three transmembrane domains (TMDs), a short N-terminal glycosylated domain located in the lumen, and a long C-terminal tail in the cytoplasm (Fig. 1B) (12, 22). Two conformers of M were reported for MHV and SARS-CoV-1. The first is called M_compact_ and is described as an indistinct ellipsoid. The second one is M_long_, as it is 2 nm longer than the former while being shaped like a dagger with the blade directed toward the cytoplasm, corresponding to the endodomain tail of M. This means that M_long_ is the conformer interacting with N, which is confirmed by a conformational change from M_compact_ to M_long_ in the presence of RNPs formed by N proteins and the viral RNA, the latter being a possible catalyst of the transformation (23). Considering that SARS-CoV-1 M presents about 90% homology (24) with SARS-CoV-2, SARS-CoV-2 M likely shares the same features.

## CONTRIBUTION OF M-M INTERACTIONS TO VIRUS ASSEMBLY

The interaction of M with itself is crucial for the assembly process, but it might not be sufficient to produce particles as the other membrane proteins E and N are also required (see below). M-M interactions in virus-like particle (VLP) production were extensively studied in the case of MHV using large amounts of M mutants. It was reported that M-M interactions are crucial and happen through multiple contact sites, creating a structure that selectively excludes foreign proteins from the membrane (25). The viral envelope appears striated because of the presence of M, and according to the estimated size of M, a maximum of four M proteins are able to make this pattern, meaning that M can be mono-, di-, tri-, or tetrameric, much like wild-type MHV (26). The carboxy-terminal tail of M seems to mediate the lateral interaction of M-M (22). This is especially the case for the SWWSFNPETNNL domain of the C terminus of M, where minor changes were not tolerated during MHV VLP formation (22). Observations of MHV particles using cryo-ET highlight an increased thickness of the inner leaflet of the membrane, corresponding to the association between the COOH-terminal tail of M and the inner leaflet (26). Additionally, the TMD interactions take part in viral spike protein (S) incorporation via M-S interactions, as explained below (25, 27).

As for SARS-CoV-1, M appears as dimers interacting with their cytosolic domain as well (23). However, according to previous research, the C terminus of M is more significant for particle formation than TMDs. Indeed, after the deletion of various domains in M, namely, the N terminus, the C terminus, or the three transmembrane segments, leaving only the C terminus, the release of vesicles was observed only in the first instance (28). Moreover, SARS-CoV-1 M and N tails interact with one another via their highly hydrophilic and charged carboxy groups, resulting in an electrostatic interaction (20) and the incorporation of the N-RNA complex into the particle (23). With SARS-CoV-2 presenting similar features, Yuan et al. highlighted the involvement of the carboxy and amino termini rather than the TMDs (29) in M-M interactions during particle formation. Overall, the carboxy-terminal tail of M is very sensitive to mutation/deletion when forming VLPs, making this extremity the most important domain involved in coronavirus assembly and particle formation (27–29). Thus, the M-N–RNA complex interaction most probably stabilizes the M tail and has been shown to enhance the production of VLPs (30, 31), probably by keeping the M protein in a favorable conformation needed for optimal particle assembly. These observations are mostly applicable to SARS-CoV-2, but the requirement and function of N, or N-RNA, in particle production are still under debate.

## IS THE INTRACELLULAR LOCATION OF M DETERMINING WHERE ASSEMBLY OCCURS?

Viral assembly and budding of coronavirus particles take place at the intermediate compartment between the endoplasmic reticulum and the Golgi apparatus (ERGIC) (Fig. 2). Thanks to tagged proteins, it was shown that SARS-CoV-1 M is located in the Golgi apparatus, when expressed alone (32). Indeed, M proteins can undergo maturation leading to N-glycosylated M proteins found in infected cells, which shows that some M proteins are modified after their translation, into the Golgi apparatus, where they mainly accumulate; some M is also found in vesicles along the secretory pathway (32). In the case of MHV, M can form clusters in the Golgi apparatus and interacts with O-glycosylated M (25). This has also been observed in the case of SARS-CoV-2, revealed by two specific bands for M proteins using immunoblotting (33). Thus, this means that coronavirus M is recycled from the Golgi apparatus to the early compartment of virus assembly, i.e., at the ERGIC, to assemble with the other structural membrane proteins E and S. This might be due to an undefined role of N-glycosylated M or to the need for having an unsaturated Golgi membrane in order to egress through the cell secretory pathway (32). Since M, E, and S show different locations when expressed alone, their coordination during particle assembly and then egress is required and might involve other viral or cellular cofactors. Extended studies are needed in order to prove or refute this theory in the case of SARS-CoV-2.

## THE ENVELOPE PROTEIN E PLAYS KEY ROLES IN PARTICLE ASSEMBLY

In coronaviruses, protein E is a small transmembrane protein of 8 to 12 kDa, composed of 75 amino acids, with a luminal short N terminus of 7 to 12 amino acids; a large hydrophobic transmembrane domain of around 25 amino acids, containing at least one amphipathic alpha-helix involved in the formation of an ion-conductive pore; and, finally, a long hydrophilic carboxyl terminus (12, 34). E is mainly oriented with its N terminus in the lumen of the ERGIC and its C-terminal tail in the cytoplasm (Fig. 1C) (35). For SARS-CoV-1, a second structure of the β-hairpin for the tail of E has been predicted and seems to be significant in locating E in the Golgi apparatus. Indeed, when the motif responsible for the formation of this secondary structure is deleted, the Golgi location of E is disrupted. When the mutation is engineered elsewhere in the tail, the location is preserved (36). This is coherent with MHV and SARS-CoV-2 E being located in the ERGIC and the Golgi apparatus, both in *cis* and in medial cisternae (35, 37). Surprisingly, another recent study located SARS-CoV-2 E at the ER, when E was tagged at the C terminus and expressed alone (38). First, as extremities are very important for interactions with other structural proteins involved in virus assembly, tagging the C terminus would in all probability have led to a mislocated protein (36). Thus, internal tagging seems more adequate to conserve the integrity and location of the E protein (37). In addition, E interacts not only with structural viral proteins but also with host proteins such as PALS1, a tight junction-associated protein, or syntenin, which impacts subcellular trafficking, even though the latter is debated (37, 39, 40). Expressing it alone could thus compromise its correct location.

All posttranslational modifications of E are still unknown, as are their roles in E incorporation, interactions with the ER/ERGIC/Golgi membranes or self-oligomerization, and consequences for viral assembly (39) (Fig. 2).

## VIROPORIN E IN PARTICLE RELEASE

Among its numerous functions, the E protein of coronaviruses is able to self-oligomerize to create a pentameric ion channel, making this protein a viroporin (37, 39, 41–43). However, it is still debated whether this ion channel is specific for Ca^2+^, Na^+^, K^+^, or H^+^ (41, 42) and if ion flux would trigger the release of the viral particles. Even though the precise region of the TMD involved in ion channel formation has not yet been identified for SARS-CoV-2, MHV E TMDs are involved in E oligomerization. Additionally, MHV E TMDs seem to have an impact on the release of viral particles, but it could be a direct (ion channel flux) or indirect (membrane fission) role of the viroporin channel or both (43).

Therefore, it is interesting to compare the envelope protein E of SARS-CoV-2 and the M2 protein of influenza viruses. M2 is also a viroporin able to form an ion channel impacting multiple stages of the influenza viral life cycle (44). We can wonder whether E has abilities similar to those of M2 during viral release, altering membrane cholesterol and inducing membrane scission and particle budding in an independent ESCRT process (45). Indeed, the M2 protein has an amphipathic helix in its cytoplasmic tail, which is intricately involved in membrane scission (46). This type of helix is also found and located in the TMDs of several coronavirus E proteins, strongly suggesting that virus particle bud scission might be one of E’s roles (42). Further studies are needed to fully understand the function of the pores created by E regarding viral particle assembly and especially viral particle budding for SARS-CoV-2.

## E-M INTERACTIONS ARE ESSENTIAL FOR PARTICLE BUDDING

It is accepted that M and E are critical for the formation of viral protein-containing vesicles for SARS-CoV-1 (5, 28, 47) and SARS-CoV-2 (29–31, 48, 49). Indeed, it has been shown that the deletion of the C termini of both SARS-CoV-2 M and E decreases their ability to interact, while the N terminus of M undergoes ubiquitination, which helps stabilize the M-E interaction (29), demonstrating the existence of ongoing connections, via their C termini and the N terminus of M. Only recently, this study showed the clear interaction between M and E for SARS-CoV-2, revealing that the M-E interaction mediates vesicle release through the enhancement of M self-interactions (29). This observation may be linked to the role of M in assembly and/or to the action of E in membrane scission. Moreover, a study demonstrated the possibility of functionally replacing MHV E by replacing it with avian infectious bronchitis virus or SARS-CoV-1 E (50). Taken together, these results underline a specific but universal interaction between E and M proteins of betacoronaviruses during particle assembly.

In a transfected-cell model, E and N were also found to interact with each other through their C termini, and the deletion of a part of E’s C terminus impacted assembly and the E-N interaction. Even though E and N might interact in the wild-type virus, the purpose of this interaction is not clear yet (51), but it may be to enhance the incorporation of RNPs, confirming the crucial role of E in assembly (37). A mutation of SARS-CoV-1 with a deletion of E showed aberrant virions, described as vesicles containing dense and granular material, with totally different features from those of a wild-type virion (52).

Taken together, these results show that E has a fundamental but still unclear role in viral assembly and particle release.

## IS THE SPIKE PROTEIN REQUIRED FOR PARTICLE ASSEMBLY?

As the incorporation of the spike protein (S) is likely mediated by other structural viral proteins during the assembly of a new particle, it is relevant to question whether S is an actor in viral particle assembly or budding. First, S is a transmembrane glycoprotein assembled as a trimer at the virus membrane and is needed for viral entry by fusion upon infection of new targeted cells. S is composed of two subunits, which are S1 and S2 (Fig. 1D) (12). S1 contains the N terminus and the receptor binding domain (RBD), which will interact with the receptor ACE2, as well as coreceptors, for viral entry (53). S2 is composed of a transmembrane domain and a C-terminal cytoplasmic tail needed for fusion (12). The region between S1 and S2 contains an arginine-rich motif, RRAR, present in SARS-CoV-2 but not in SARS-CoV-1. It is cleaved by the host protein furin during spike maturation at the ER/Golgi apparatus (54, 55). This cleavage acts as a preactivation of the spike and induces a conformational change, unmasking the RBD required to recognize ACE2 (55, 56). It is clear that this cleavage is essential for SARS-CoV-2 infectivity (56) and is probably involved in cell-cell and virus-cell transmission (54). The result is a cleaved spike with S1 and S2 interacting with one another through noncovalent interactions (55). Near the cleavage motif, an O-glycopeptide is present and might regulate furin-dependent cleavage (57). The role of transmembrane protease serine 2 (TMPRSS2), also involved in spike maturation needed for viral entry, is still uncertain. Indeed, a recent study demonstrated that TMPRSS2 is not directly involved in S1/S2 or S2′ cleavage, the latter being a motif located near the S2 fusion peptide (55) (Fig. 1D). However, TMPRSS2 seems to cleave another domain in S leading to a more exposed S2′ cleavage site, resulting in membrane fusion (55). The question of knowing whether the cleavage and maturation of S are involved in particle assembly or release in producer cells is still pending.

The SARS-CoV-2 spike protein is also widely glycosylated, with 22 N-linked glycan sites (58) and 17 N-glycosylation motifs identified (57). Posttranslational modifications such as folding and glycosylation and proteolytic processing of the spike seem to have a crucial role in the infectivity and assembly of newly formed particles (58). Indeed, S needs to be N-glycosylated in order to be incorporated into the particle (27, 59). For SARS-CoV-2, it has been reported that once posttranslational modifications are achieved, small *trans*-Golgi transport vesicles supply spike proteins to the ERGIC via membrane fusion. It seems that S is embedded in the ERGIC membrane and then clusters when the N-RNA complex is incorporated into a newly formed particle, likely through M-S interactions (3) (Fig. 2). Additionally, S trimers undergo reorganization at the ERGIC membrane and spread over the viral particle during the assembly process driven by M (7). For SARS-CoV-1, both glycosylated M and M are able to interact with S (23), most probably through TMD interactions between M conformers and S, as was reported in the case of MHV (27). The lack of S incorporation in M-free membranes strengthens the role of M in S incorporation during particle assembly (23).

Furthermore, M and E can modulate the maturation and location of S by keeping it inside the cell and avoiding its expression at the cell surface, as was recently reported for SARS-CoV-2 (30). Indeed, in the absence of these two main structural proteins, S is present at the cell plasma membrane, as shown by the formation of syncytia in cells when S is expressed alone. This creates large multinucleated cells similar to some SARS-CoV-2-infected cells, likely due to the fusion ability of S (30, 60). When M and E are present, S is located within the Golgi apparatus, especially the *cis*-Golgi, and also near the ERGIC. Moreover, S seems to be retained by M via its carboxyl terminus in order to be recycled from the cell membrane (30). Similar to SARS-CoV-1, SARS-CoV-2 S might also interact with coatomer complex I (COPI) in order to be recycled from the Golgi apparatus, possibly increasing M-S interactions (61).

Additionally, if SARS-CoV-2 E slows the cell secretory pathway, therefore inducing the global retention of glycoproteins inside the cell, such as M or S, for instance, it might have an indirect effect on retaining S inside the cell (30). Indeed, when the C-terminal tail, responsible for its M-mediated intracellular retention, is removed from the SARS-CoV-2 S protein and is expressed in the presence of E, a similar kind of retention is observed. This intracellular retention may be carried out in order to concentrate viral structural proteins at the ERGIC to favor viral particle assembly (30). The contribution of one or several host cofactors to this process or a direct undescribed interaction between S and E needs to be investigated.

Altogether, these results tend to indicate that the spike protein is not directly involved in viral particle formation (i.e., membrane bending or budding), although it could be responsible for stabilizing the budding particle or simply may have an impact on virus assembly kinetics.

## MINIMAL REQUIREMENTS OF VIRAL STRUCTURAL PROTEINS FOR VLP FORMATION

Even though coronaviruses share some features in particle assembly and release, others can diverge. For instance, the minimal required combination of structural proteins needed to release particles can differ, but optimal VLP production requires M, N, and E in the case of SARS-CoV-1 (5, 28) and SARS-CoV-2 (29–31, 48, 49), with or without S. Indeed, SARS-CoV-1 M and N are necessary and sufficient for forming vesicles containing M and N, but the addition of E increases the production and release of VLPs (28). However, a continued increase in the amount of E does not affect the overall production yield (5), suggesting an unexplained steric constraint on E incorporation into the VLP or strong regulation of E expression during virus replication. This conclusion is confirmed by a study using a bicistronic vector that expresses M and E together. The total amount of E produced is thereby very small, similar to the wild-type virus composition (5). Thus, M, N, and E appear to be the minimal required combination of structural viral proteins to form a VLP. Nevertheless, a study on Sf9 insect cells with baculovirus infection showed that M and E are sufficient to form SARS-CoV-1-derived particles, whereas N is not incorporated (47). This might be linked to the nonmammalian cell type system, which does not confer the correct protein glycosylation pattern and therefore mitigates the conclusion of this study. As for MHV, the budding process is N independent, requiring only the membrane proteins M and E (62).

For SARS-CoV-2, minimal requirements for particle formation vary depending on the studies: it seems to rely on S, E, and M to produce competent VLPs in HEK293T cells (31, 49). Other studies show that only M and E appear to be needed as a minimal system of vesicle formation in Vero E6 and HEK293T cell lines (29, 48). However, for the purpose of having optimal VLP morphology and production, all the structural proteins of SARS-CoV-2 (Fig. 1) seem to be required (30, 31). Indeed, M and E are needed for ERGIC retention and the maturation of N-glycosylated S proteins, prior to incorporation into VLPs. Overall, recent studies highlight the fact that SARS-CoV-2 VLP assembly called for well-balanced quantities of all structural proteins and that the M-E couple seems to be the driving force of particle formation and necessary for VLP production, regardless of the cell type.

## M-E COUPLE: WHICH ONE DRIVES MEMBRANE BENDING?

The recruitment of several structural viral proteins at the site of virus assembly raises the question of whether any of them are involved in membrane curvature during particle formation. E can be considered a candidate as it remains located at the ERGIC (35), the site of particle assembly and budding. Indeed, it has been reported that the MHV E protein is able to create tubular structures at the assembly site, which may help to curve membranes during particle formation (36). For SARS-CoV-2, a monomeric E structure and dynamic simulation suggest that the amphipathic helices of the C terminus of E are engaged in membrane bending (63). Overall, this is in favor of E having a role not only in membrane scission for particle release but also in priming membrane bending. Concerning the role of the M protein in membrane bending during assembly, the network created by M-M interactions could also be involved in membrane curvature thanks to the energy or asymmetry provided by these interactions (25). Overall, the association of M and E appears to be required for triggering membrane bending during particle formation, without considering that other cellular factors might be involved.

## ARE OTHER STRUCTURAL PROTEINS INVOLVED IN MEMBRANE CURVATURE?

For SARS-CoV-1, it appears that the switch between M conformers, due to the M-N interaction, plays a key role in membrane curvature because only M_long_ seems to form a convex and rigidified viral envelope. Nevertheless, it is not completely clear whether the N-RNA complexes trigger membrane curvature once attached to the M tail or whether the RNPs accumulate near the already curved membrane regions (23) induced by M, E, or the M-E association. As for the spike protein, cryo-ET studies on SARS-CoV-2 exhibited no positive bending of the ERGIC where S was accumulated (7). This indicates that S alone is unable to initiate membrane bending and *a fortiori* viral budding, which is consistent with the fact that S is not required for VLP release. Finally, it cannot be ignored that cell-associated cofactors, such as proteins or lipids, might be needed in order to help viral membrane bending or membrane scission during SARS-CoV-2 particle formation, such as the E3 ubiquitin ligase RNF5 that was recently shown to reinforce M-E interactions for mediated virus release (29).

## SEVERAL EGRESS PATHWAYS FOR CORONAVIRUS PARTICLES?

Once viral particle assembly and budding occur at the ERGIC membrane and in its lumen, respectively, newly made viral particles will be transported into intracellular virus-containing vesicles toward the plasma membrane to be released into the extracellular medium (Fig. 2). Viruses egress either through single-membrane vesicles (SMVs), when there are few particles, or through large virus-containing vesicles (LVCVs), loaded with multiple viruses. The exact provenance and nature of such vesicles (3) have yet to be discovered.

The kind of egress of SARS-CoV-2 seems to differ between these two types of transport vesicles. The particles contained in LVCVs likely egress via exocytosis. Indeed, cryo-ET shows viruses exiting tunnels, likely resulting from membrane fusion between the cell membrane and LVCV. As for SMVs, several observations link the presence of only one virus outside the cell and plasma membrane discontinuities. This could be the result of another egress pathway accompanied by membrane rupture (3). These observations are in agreement with a previous study on VLPs from SARS-CoV-1 structural proteins. Siu et al. pointed out three types of fluorescent objects when N was tagged with green fluorescent protein (GFP). The first type corresponded to large static vesicles near the nucleus, possibly indicating the assembly site. The second type consisted of smaller vesicles characterized as very dynamic, while the last one referred to very small vesicles, probably containing single particles or released VLPs. These results were also confirmed by electron microscopy (EM) in VLPs and in wild-type SARS-CoV-1 (5). These transport vesicles containing SARS-CoV-1 particles were identified as part of the cell secretory pathway thanks to the use of brefeldin A (BFA). Among other things, this metabolite affects Golgi integrity and the secretory pathway by inhibiting traffic from the ER to the Golgi apparatus. For SARS-CoV-1 VLPs, this addition leads to the disruption of the above-described vesicles and alters VLP transport. This strongly suggests that the secretory pathway is the main exit route used by SARS-CoV-1 (5). Nevertheless, BFA addition does not hamper the release of new viral particles in wild-type MHV, meaning that other exit pathways may be used by coronaviruses. Indeed, MHV egresses via lysosomal trafficking despite the secretory pathway being operational. The lysosome-associated membrane protein 1 (LAMP-1) level increases in infected cells and colocalizes with MHV particles, suggesting that the endolysosomal pathway can be used for virus egress (64). This is in accordance with EM images showing that in the case of SARS-CoV-2, intracellular viral particles are contained in organelles displaying all the lysosome characteristics as for MHV (64). Additionally, the number of acidified lysosomes decreased upon SARS-CoV-2 and MHV infection, and the lysosomes were deacidified by an average of 1 pH unit in MHV-infected cells (64). This may be due to either a high number of particles inside the lysosome, overfilling it, or a disturbance of the proton pump caused by any of the viral proteins. Indeed, SARS-CoV-2 open reading frame 3a (ORF3a), which has been reported to be a viroporin, is located in the late endosome and might play the role of a proton pump in order to deacidify the lysosomes and create a better environment for viral transport (38, 64). In addition, a recent study showed that an increase of E proteins at lysosomes causes a decrease in their acidity (37). The consequence of this higher pH is a strong decrease in lysosome enzyme activity (64) that certainly prevents virus degradation. Simultaneously, it has been shown that when SARS-CoV-2 M is ubiquitinated, it induces autophagy and regulates the degradation ability of the lysosome in order to likely use it as an escape route for viral particles (29). Altogether, these results strongly suggest that unresolved mechanisms are affecting vesicular trafficking upon infection for coronaviruses to egress, and this deserves further studies.

An interactive protein-protein map between SARS-CoV-2 proteins and host cell proteins was recently reported and strongly suggests that several membrane trafficking proteins, such as Rab7, are involved in close association with viral nsp7 (21). Thus, another complementary pathway would be the use of exosomal biogenesis by SARS-CoV-2. As evidence, the above-mentioned protein map exhibits exosomal biomarkers with nsp8 upon cell line infection (21), suggesting that part of SARS-CoV-2 egresses through the exosomal pathway, potentially in a comparative manner, as was described for other enveloped viruses such as human immunodeficiency virus type 1 or hepatitis viruses (65–67).

## CONCLUSIONS ON SARS-CoV-2 PARTICLE ASSEMBLY AND RELEASE

By assuming common points between SARS-CoV-2 and its most closely related betacoronaviruses, SARS-CoV-1 and MHV, here, we propose features for SARS-CoV-2 particle genesis, assembly, and budding (Fig. 2). First, the viral genomic RNA is synthesized inside DMVs and released into the cytoplasm through membrane pores. Upon its exit, it is probably directly complexed with abundant cytosolic N proteins, consequently forming a beads-on-a-string structure containing 30 to 35 beads of RNPs that have been recently imaged using transmission electron microscopy (TEM) (7). However, uncertainty lies in the number of N proteins per RNP. Once the composed N-RNA complexes are formed in the cytosol, a liquid-phase change certainly occurs (11, 15, 19) that could regulate RNP assembly on ERGIC membranes. This regulation might also involve other unknown viral regulators or host factor interactions required for coronavirus assembly that need to be discovered.

One hypothesis (30) states that the viroporin E could retain all the structural proteins in the ERGIC membranes, creating a local concentration of the structural viral proteins E, M, and S needed for particle assembly. Glycosylated structural proteins would be transported, probably together, from the Golgi apparatus to the ERGIC to be incorporated into the nascent particle by an unknown mechanism, passive or active. The sequence and motifs located in these proteins for structural protein clustering into the ERGIC also remain to be further studied. On the other hand, thanks to many N-RNA and M-N interactions, the complex containing the RNA is compacted to a certain degree and then incorporated into the nascent budding particle containing M, E, and S (Fig. 2) (17, 19). M could then be responsible for membrane bending through M-M interactions in order to favor particle budding inside the ERGIC, likely with the contribution of E and by a reported change in the conformation induced by the interaction with N (23). This hypothesis remains to be proven, and in that sense, molecular modeling could help (68). However, a question remains: since VLPs or vesicles can be formed without N, it lets us wonder if E is the major structural protein responsible for the membrane bending process or if M is required or even sufficient. Thereafter, the mechanisms by which the scission of the viral bud occurs in the ERGIC can occur through the action of either E (comparable to M2 of influenza viruses, where it alters membrane cholesterol, leading to membrane curvature and scission thanks to an amphiphilic helix [45, 46]) or an undescribed ESCRT-dependent or -independent process that is yet to be discovered. Scission could also be facilitated by specific lipid recruitment or by the assistance of unknown cellular cofactors.

Finally, once the ERGIC contains a single or several particles inside, vesicles containing viral particles apparently progress through the secretory pathway, and viruses egress. An alternative exit has been proposed through the exosomal pathway (65) and/or using the lysosomal pathway via deacidification (64). It can be assumed that this decreased acidity is due to the high number of particles located inside these lysosomes or because of, for example, the viral protein E or Orf3a, located in the late endosomes, which can form a cation channel as a proton pump. Some exosomal markers have been found associated with released particles (21), while others report the implication of the lysosomal or autophagy pathways (64), making the egress pathway(s) of SARS-CoV-2 difficult to understand. Further investigations are needed to distinguish these pathways and explain if they are fused, dysregulated, or highjacked upon infection, as was reported for other enveloped RNA viruses (65–67). In conclusion, the evidence available to correctly or completely describe SARS-CoV-2 egress from infected cells is incomplete and even more so from polarized pulmonary host cells and more relevant cell biology models, leaving a wide field of research interest in elucidating the mechanisms of SARS-CoV-2 particle formation, budding, and egress.
